# Mutant huntingtin induces iron overload via up-regulating IRP1 in Huntington’s disease

**DOI:** 10.1186/s13578-018-0239-x

**Published:** 2018-07-04

**Authors:** Li Niu, Cuifang Ye, Yun Sun, Ting Peng, Shiming Yang, Weixi Wang, He Li

**Affiliations:** 1 Department of Histology and Embryology, School of Basic Medical Sciences, Tongji Medical College, Huazhong University of Science and Technology, 13 Hangkong Road, Wuhan, 430030 People’s Republic of China; 2 Institute for Brain Sciences, Tongji Medical College, Huazhong University of Science and Technology, Wuhan, 430030 People’s Republic of China; 3 Collaborative Innovation Center for Brain Science, Huazhong University of Science and Technology, Wuhan, 430030 People’s Republic of China

**Keywords:** Huntington’s disease, Iron, Iron response protein 1, Transferrin receptor, Transferrin, Ferritin

## Abstract

**Background:**

Iron accumulation in basal ganglia accompanies neuronal loss in Huntington’s disease (HD) patients and mouse disease models. Disruption of HD brain iron homeostasis occurs before the onset of clinical signs. Therefore, investigating the mechanism of iron accumulation is essential to understanding its role in disease pathogenesis.

**Methods:**

N171-82Q HD transgenic mice brain iron was detected by using Diaminobenzidine-enhanced Perls’ stain. Iron homeostatic proteins including iron response protein 1 (IRP1), transferrin (Tf), ferritin and transferrin receptor (TfR) were determined by using western blotting and immunohistochemistry, and their relative expression levels of RNA were measured by RT-PCR in both N171-82Q HD transgenic mice and HEK293 cells expressing N-terminal of huntingtin.

**Results:**

Iron was increased in striatum and cortex of N171-82Q HD transgenic mice. Analysis of iron homeostatic proteins revealed increased expression of IRP1, Tf, ferritin and TfR in N171-82Q mice striatum and cortex. The same results were obtained in HEK293 cells expressing N-terminal of mutant huntingtin containing 160 CAG repeats.

**Conclusion:**

We conclude that mutant huntingtin may cause abnormal iron homeostatic pathways by increasing IRP1 expression in Huntington’s disease, suggesting potential therapeutic target.

## Background

Huntington’s disease (HD) is an autosomal dominant neurodegenerative disease caused by a pathological expansion of CAG repeats (> 36) in the first exon of the gene encoding huntingtin [1]. Striatum and cerebral cortex of HD patients undergo gradual degeneration starting several years before clinical onset [2]. Mutant huntingtin (mHTT) leads to multiple detrimental outcomes including abnormal gene transcription [3–6], oxidative stress [7], mitochondrial dysfunction [8], altered calcium homeostasis [9]. Additionally, iron overload might contribute to HD onset [10–12]. Iron accumulation naturally occurs in all aging mammals, and it is involved in cognitive and motor dysfunction in the elderly [13]. Iron is increased in the neurodegenerative brain areas in HD, and iron homeostasis is disrupted in the disease [10, 14, 15]. Disruption of brain iron homeostasis in HD patients occurs before the onset of clinical signs [15, 16] which suggests that iron is closely involved in the mHTT-induced pathological cascade [17, 18].

In mammals, cellular iron homoeostasis is largely coordinated at the post-transcriptional level through the action of two cytoplasmic iron regulatory proteins (IRP1 and IRP2). They are RNA binding proteins that respond to cellular iron levels and post-transcriptionally bind to mRNA stem loop structures known as iron-responsive elements (IREs) based on cellular iron concentrations. IRPs function by binding to IREs in the mRNAs that code for ferritin and transferrin receptor (TfR) [19–21]. TfR is an important protein that facilitates receptor-mediated endocytosis of the iron carrying proteins transferrin (Tf) [19, 21]. Tf is the main extracellular iron carrier, with two binding sites for ferrous iron. Iron-Tf/TfR is internalized by receptor-mediated endocytosis and trafficked to endosomes, where iron is liberated from Tf to leave the endosome. Imported iron is used directly, incorporated into heme or Fe-S clusters, or stored in ferritin. When cellular iron is deficient, IRPs will bind IREs on ferritin mRNAs to block translation of this iron storage protein, and TfR mRNAs are stabilized to promote iron uptake. Conversely, in iron surfeit, ferritin mRNAs are actively translated to store excess iron, and mRNAs encoding iron import-related proteins are degraded [17]. Cells can regulate their iron content by the IRE/IRPs homeostatic mechanism [17]. Although both IRP1 and IRP2 bind consensus IREs with high affinity [22], their regulation model is different. The regulation of IRP2 activity is mediated by iron-induced degradation of the protein [19, 22]. In contrast to IPR2, IRP1 is stable and its function as an RNA-binding protein is determined by the reversible assembly of Fe-S cluster [19]. When cells are iron-replete, IRP2 is rapidly degraded by the ubiquitin proteasome and is therefore not able to bind IREs [20, 22], whereas IRP1, though still present, no longer play a function of an RNA binding protein [21]. Remarkably, a single IRP, IRP1, can regulates expression of both ferritin and the TfR in vivo [21].

It has been reported that several iron homeostatic proteins including IRP1 and IRP2 contribute to neurodegenerative disease processes [14, 23]. Huntingtin knockdown in zebra fish results in an iron deficiency phenotype during development [24], and deletion of the *huntingtin* gene increases significantly the expression of iron uptake protein TfR [24], suggesting that huntingtin is implicated in iron homeostasis. Given that iron does not interact with N-terminal huntingtin fragments, the most active region of the protein [25, 26], we presume that mHTT affects iron level by disrupting iron homeostatic pathways. To test the hypothesis, we examined iron content in the N171-82Q mice brain and effects of mHTT on iron homeostatic modulatory machinery. We found that iron is increased in the striatum and cortex of N171-82Q mice, and mHTT up-regulates the expressing of IRP1, Tf and TfR. The study provides a sight for additional therapeutic options targeted iron homeostasis in HD.

## Methods

### Mouse husbandry

B6C3-Tg (HD82Gln) 81Dbo/J (N171-HD82Q) HD transgenic mice were obtained from Jackson Laboratories. The HD mice express a cDNA encoding a 171 amino acid N-terminal fragment of huntingtin containing 82 CAG (Q) repeats. Wild-type (WT) littermates were used as controls. Tails snips were obtained at the age of 4 weeks and mice were genotyped by PCR. Hemizygosity for the HD transgene were determined according to the Jackson Laboratories protocol. The mice had free access to food and water and were maintained under standard conditions with a 12-hour dark–light cycle at stable temperature (23–25 °C). All mouse experiments were approved by the Institutional Animal Care and Use Committee of Tongji Medical College, Huazhong University of Science and Technology, and performed in compliance with the National Institutes of Health Guide for the Care and Use of Laboratory Animals.

### Cell culture and transfection

Human Embryonic Kidney 293 (HEK293) cells were maintained in Dulbecco’s modified Eagle’s medium (DMEM) supplemented with 10% fetal bovine serum (GIBCO, Australia), 100 units/ml streptomycin, and 100 μg/ml penicillin in a humidified incubator at 37 °C under 5% CO_2_ and 95% air. Once they reached 90–95% confluence, 1 × 10^5^ cells/well was planted into 6-well plates. After 24 h, they were transiently transfected with plasmids using Lipofectamine TM 2000 (Invitrogen). At 6 h after transfection, the media was replaced with fresh culture medium according to the Lipofectamine TM 2000 protocol. The pEGFP-exon-1 HTT with 20 CAG repeats (20Q) and 160 CAG repeats (160Q) plasmid were produced in our library.

### Diaminobenzidine (DAB)-enhanced Perls’ stain

Mice were deeply anesthetized with pentobarbital sodium intraperitoneal injection (40-45 mg/kg body weight) and perfused transcardially with saline followed by 4% paraformaldehyde in 150 ml 0.1 M PBS. Their brains were carefully removed and further post-fixed with the same fixatives for 8 h at 4 °C. The samples were immersed in 30% sucrose at 4 °C for 12 h and sectioned at 30 μm using a freezing microtome.

For the Diaminobenzidine (DAB)-enhanced Perls’ stain, the brain sections from 12-week-old N171-82Q mice and control WT mice (n = 3 for each) were treated with a mixture of 5% HCl and 5% potassium ferrocyanide (1:1, V:V) for 30 min. After rinsing in deionized water for 30 min, sections were incubated in 0.5% DAB in Tris–HCl buffer (pH 8.0) for 20 min, then rinsed in deionized water for 5 min, dehydrated in graded alcohols, cleared in xylene and mounted. Images were taken on a Nikon microscope (Digital Camera DXM 1200).

### Immunohistochemistry

For immunohistochemical analysis of Tf, TfR, ferritin and IRP1, free-floating sections from 12-, 14- and 16-week-old N171-82Q mice (n = 3 at each age) and age-matched WT mice (n = 3 at each age) were immersed in a mixture of 3% hydrogen peroxide (H_2_O_2_) and 0.3% TritonX-100 for 20 min to block endogenous peroxidase activity, and were pre-incubated with 3% bovine serum albumin (BSA) to reduce non-specific staining. The sections were incubated overnight at 4 °C with polyclonal rabbit antibodies against Tf, TfR, ferritin and IRP1 (all at dilution 1:200, Proteintech) in BSA with 2% goat serum and 0.3% TritonX-100. After 24 h, the sections were washed twice with 0.01 M PBS then incubated with biotinylated anti-rabbit IgG diluted 1:200 (Jackson ImmunoResearch Laboratories) and avidin–biotin complex for 2 h at room temperature (RT). The sections were washed twice with 0.01 M PBS again, and then incubated in 0.02% diaminobenzidine (DAB, Sigma-Aldrich) and 0.005% hydrogen peroxide in 0.05 M Tris–HCl buffer for 10 min at RT. Images were taken on a Nikon microscope (Digital Camera DXM 1200).

### Western blotting

Striatum and cortex from 12-, 14- and 16-week-old N171-82Q mice (n = 4 at each age) and age-matched WT mice (n = 4 at each age) and HEK293 cells at different time after transfection (n = 4 at each time interval) were homogenized in an ice-cold lysis buffer containing 50 mM Tris (pH 8.0), 150 mM NaCl, 1% TritonX-100, protease inhibitor cocktail (Sigma-Aldrich) and 100 mg/ml phenylmethylsulfonyl fluoride (PMSF). The homogenate was centrifuged at 12,000*g* for 30 min at 4 °C. The extracted proteins were separated on 10% SDS–polyacrylamide gels and transferred onto nitrocellulose filter membranes (Amersham Biosciences UK Limited) in an electrotransfer device (90 V, 90 min). The membranes were then blocked in 5% nonfat milk in 0.01 M PBS for 1 h and incubated in primary antibody overnight at 4 °C at the following dilutions: Tf (1:1000), TfR (1:1000), ferritin (1:1000), IRP1 (1:1000) and GAPDH (1:5000, Sigma-Aldrich). The membranes were then washed with 5% nonfat milk and immersed in the horseradish peroxidase-conjugated secondary antibody for 2 h at RT with constant agitation. The immunoreactive bands were visualized by exposure to an enhanced chemiluminescence (ECL) kit (Thermo Fisher Scientific).

### RT-PCR

Striatum and cortex from 12-, 14- and 16-week-old N171-82Q mice (n = 4 at each age) and age-matched WT mice (n = 4 at each age) and HEK293 cells at different time after transfection (n = 4 at each time interval) were used for RT-PCR. Total RNA from different sample was obtained with Trizol reagent (Invitrogen). Tf, TfR, ferritin and IRP1 mRNA expression was amplified by reverse-transcription polymerase chain reaction and β-actin mRNA was taken as an internal control.

The following PCR conditions were used: 94 °C for 3 min; 30 cycles of denaturing at 94 °C for 30 s, annealing for 30 s, extension at 72 °C for 45 s, and a final extension at 72 °C for 3 min. Photos of the amplified genes were taken after agarose gel (2%) electrophoresis. The primers and amplification used in the PCRs are listed in Table 1.

**Table 1:** Primers, amplicon size and annealing temperature of Tf, TfR, Ferritin, IRP1 and β-actin for RT-PCR

Name	Primer sequences		Amplicon size (bp)	Annealing temperature (°C)
	Forward	Reverse		
β-actin	GTCGTACCACAGGCATTGTGATGG	GCAATGCCTGGGTACATGGTGG	492	58/63
β-actin	TTTCCAGCCTTCCTTCTTGGGTATG	ATAGAGGTCTTTACGGATGTCAACG	100	58/63
Tf	AAACGGTCAAATGGTGCG	CTCCGACAGCTTACAGAAGAGC	413	58
TfR	TCCCGAGGGTTATGTGGC	GGCGGAAACTGAGTATGATTGA	324	58
Ferritin	TTTGACCGAGATGATGTG	TCAGTAGCCAGTTTGTGC	248	58
IRP1	GACATCGTGCTCACCATTACCAA	TGAAATCTCGAAACATGCCTACA	265	58

### Statistical analysis

All images were analyzed by using the Image-pro Plus 6.0 Image analysis software. Data were represented as mean ± SEM from three or four independent experiments. Statistical analyses were performed using SPSS Statistics 17.0 software for one-way ANVOA followed by the Student *t* test. Differences were considered significant if *p *< 0.05.

## Results

### The N171-82Q mice exhibits a higher iron level in the brain

**Fig. 1:**
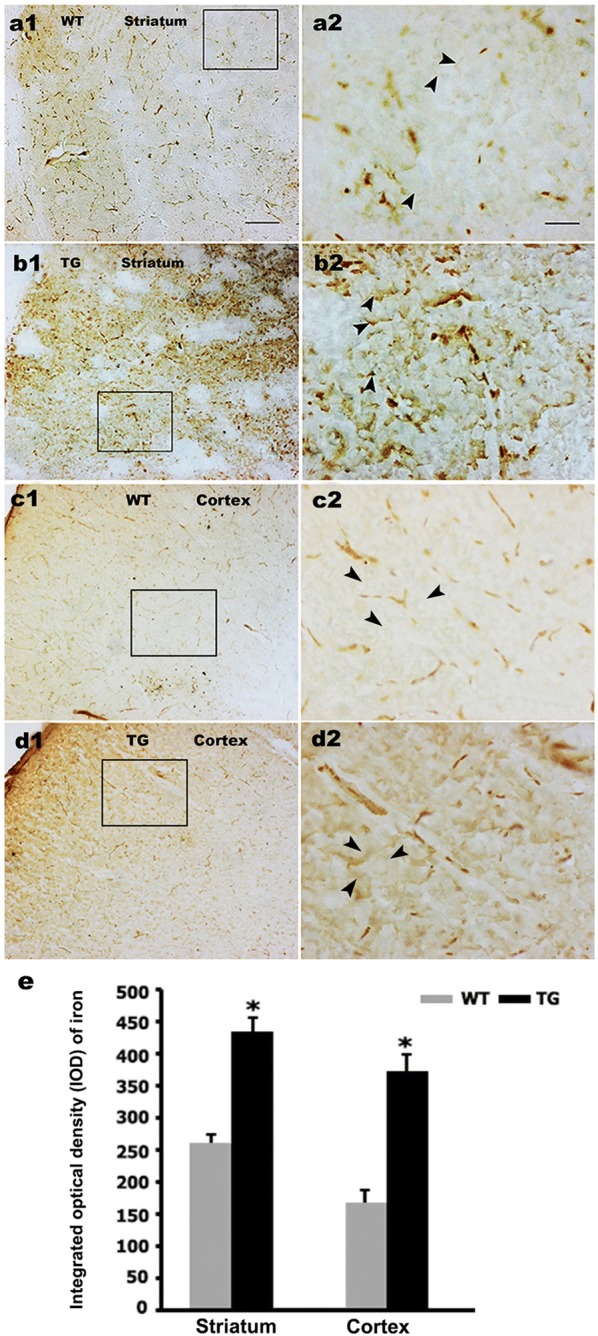
Irons levels increase in striatum and cortex of N171-82Q mice. **a**–**d** Irons levels were detected in striatum and cortex of 12-week-old N171-82Q HD transgenic (TG) mice and age-matched wild type (WT) mice by DAB-enhanced Perls’ stain. **a2**–**d2** respectively are the amplified images of squared areas in **a1**–**d1**. Arrowheads show iron deposition. Bar in **a1**–**d1** is 100 μm, and bar in **a2**–**d2** is 25 μm. **e** Integrated optical density measurement shows the difference in iron staining. Compared to age-matched WT mice, the iron levels increase in striatum and cortex of TG mice. n = 3. *p < 0.05 compared to WT mice

First, we explored the effect of mHTT on iron levels in the brain of N171-82Q mice by using DAB-enhanced Perls’ stain, the most commonly used histochemical technique for detecting iron. Under these condition ferric ion deposits are stained brown. In the 12-week-old WT mice, faint iron staining was observed in the striatum (Fig. 1a). In contrast, the age-matched N171-82Q mice striatum exhibited higher Perls’ staining (Fig. 1b, e). Similar results were observed in the cortex (Fig. 1c–e), suggesting mHTT induces a substantial increase in the brain iron level.

### IRP1 expression increases in the cortex and striatum of N171-82Q mice and 160Q HEK293 cells

**Fig. 2:**
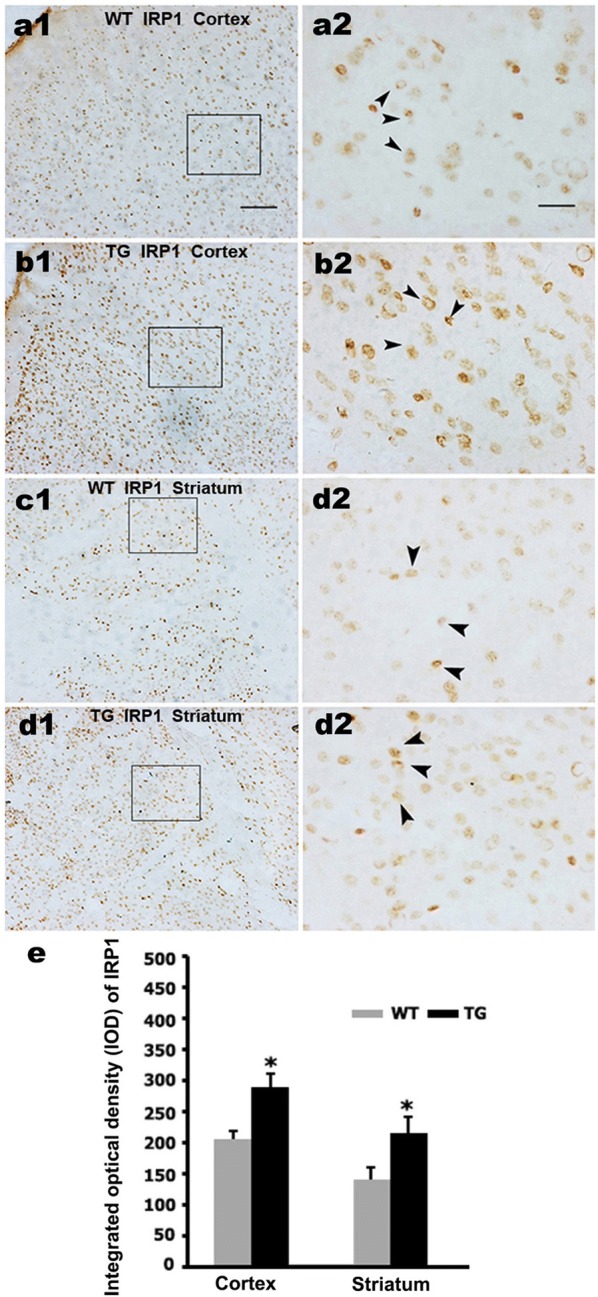
The N171-82Q mice displays dense IRP_1_-immunoreactivities in striatum and cortex. **a**–**d** IRP1 expression was detected in cortex and striatum of 12-week-old TG mice and age-matched WT mice by immunohistochemistry. **a2**–**d2**, respectively are the amplified images of squared areas in **a1**–**d1**. Arrowheads show positive IRP1 staining. Bar in **a1**–**d1** is 100 μm, and bar in **a2**–**d2** is 25 μm. **e** Integrated optical density measurement shows the difference in IRP1 immunoreactivity whose expression is significantly increased in cortex and striatum of TG mice. n = 3. *p < 0.05 compared to WT mice

Iron homeostasis is tightly controlled by IRPs. IRP1 is the most abundant isoform that can bind to mRNAs bearing IREs and decrease expression of iron-related proteins such as TfR and ferritin [27, 28]. Previous studies indicated that mHTT interferences various genes expression [4–6]. Thus, we first examined the expression of IRP1 in N171-82Q HD mice brain. Immunohistochemistry staining showed that IRP1, stained brown and black in the nucleus, exhibited denser immunoreactivities in the 12-week-old N171-82Q mice cortex (Fig. 2b, e) and striatum (Fig. 2d, e), whereas age-matched WT mice displayed only weak immunoreactivities in the same brain regions (Fig. 2a, c).

**Fig. 3:**
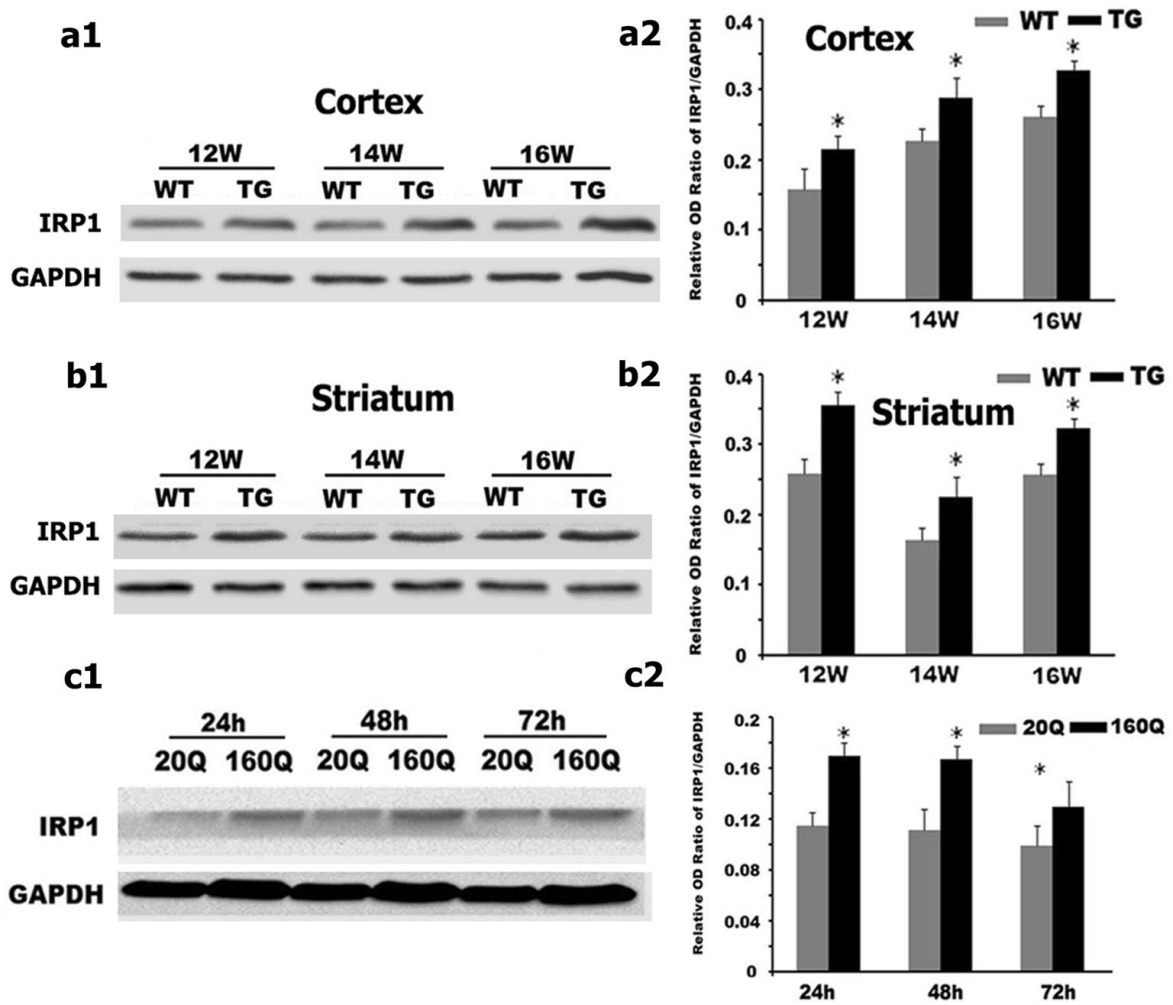
IRP_1_ protein level is increased in both N171-82Q mice brain and 160Q HEK293 cells. **a1**–**c1** IRP_1_ protein expression was detected by Western blotting both in the brain of TG mice (**a1** cortex; **b1** striatum) and in 160Q HEK293 cells (**c1**). **a2**–**c2** Quantitative representation of IRP_1_ band intensity normalized to GAPDH. IRP_1_ protein level is increased both in TG mice brain and in 160Q HEK293 cells compared to controls. n = 4. *, *p *< 0.05 compared to WT mice, or 20Q HEK293 cells

**Fig. 4:**
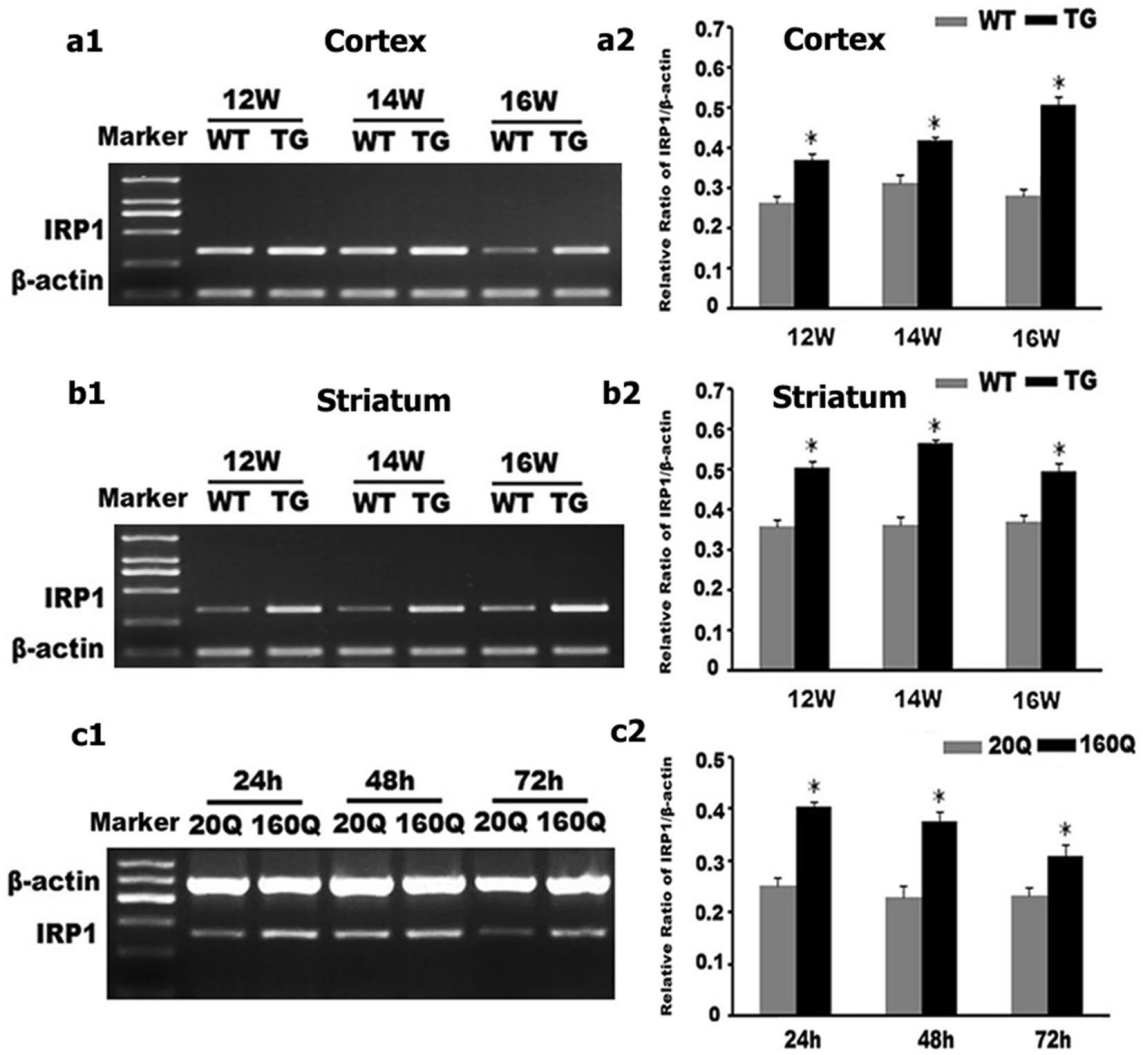
IRP_1_ mRNA level is increased in both N171-82Q mice brain and 160Q HEK293 cells. **a1**–**c1** IRP_1_ mRNA level was detected by RT-PCR both in the brain of TG mice (**a1** cortex; **b1** striatum) and in 160Q HEK293 cells (**c1**). **a2**–**c2** Quantitative representation of IRP_1_ band intensity normalized to β-actin. IRP_1_ mRNA level is increased both in TG mice brain and in 160Q HEK293 cells compared to controls. n = 4. *p < 0.05 compared to WT mice, or 20Q HEK293 cells

Further, by using western blot and RT-PCR, we found that IRP1 protein and mRNA levels in both cortex and striatum were higher in 12 to 16 weeks old N171-82Q mice compared to age-matched WT controls (Figs. 3a, b, 4a, b).

We next examined IRP1 expression in HEK293 cells expressing N-terminal of huntingtin containing 160 polyglutamine (160Q HEK293 cells) or 20 polyglutamine (20Q HEK293 cells). Consistent with the findings in the N171-82Q mice, we identified a significant increase in IRP1 protein and mRNA levels in 160Q cells compared to 20Q cells (Figs. 3c, 4c).

When iron is deleted in the cell, IRP1 will function in its IRE-binding role to prevent initiation of ferritin translation and inhibit degradation of the TfR transcript [19]. Here, the N171-82Q HD mice, though iron overloaded, displayed a higher expression of IRP1. These results indicated that the increased IRP1 expression in N171-82Q mice and 160Q HEK293 cells might be one factor to iron accumulation.

### mHTT up-regulates the expression of TfR, Tf and ferritin in the N171-82Q mice and 160Q HEK293 cells

**Fig. 5:**
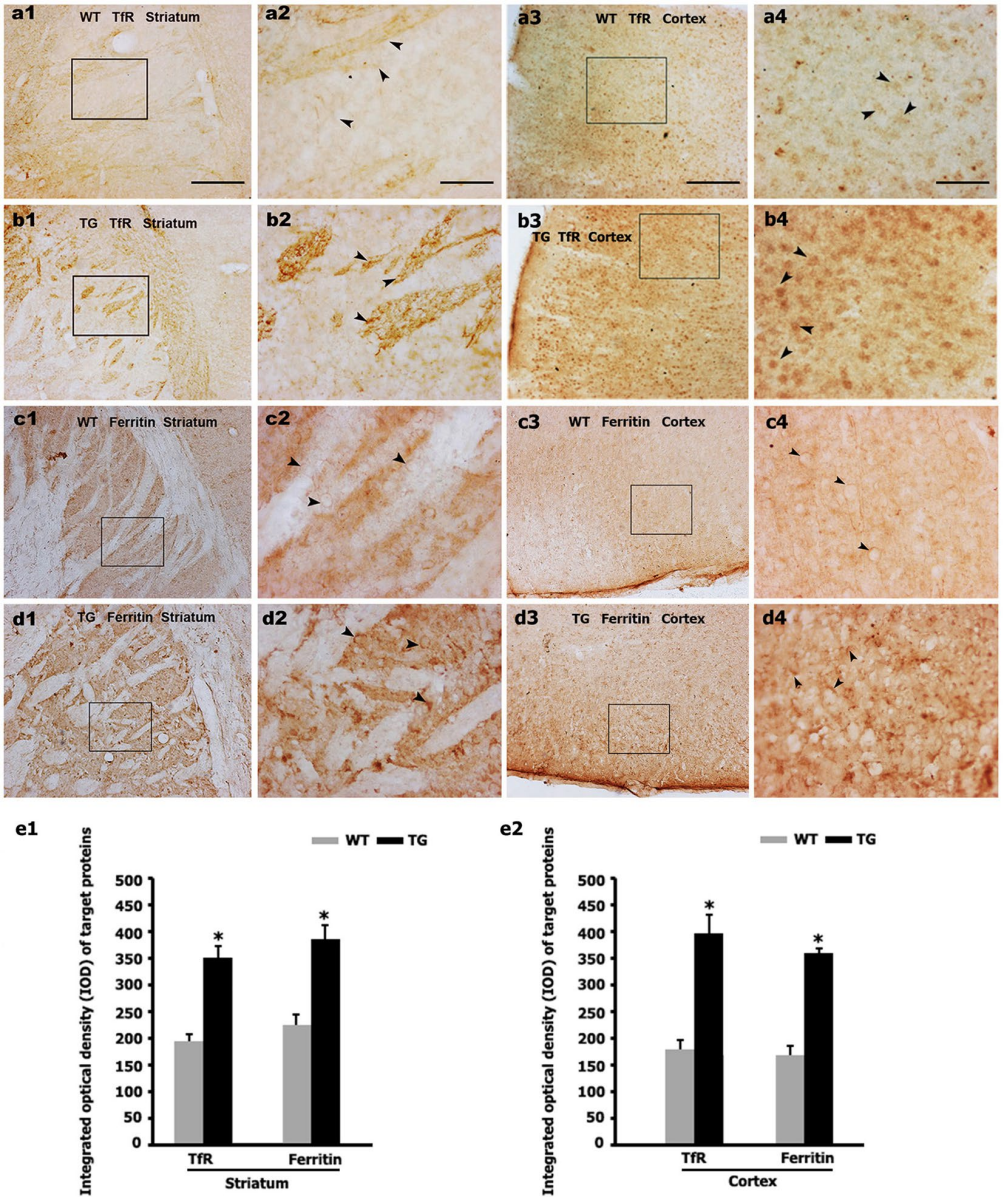
The striatum and cortex of N171-82Q mice display dense TfR and ferritin immunoreactivities. **a**–**d** The expression of TfR (a and b) and ferritin (**c**, **d**) was detected by immunohistochemistry in striatum and cortex of 12-week-old TG mice and age-matched WT mice. **a2**–**d2**, respectively are the amplified images of squared areas in **a1**–**d1**. **a4**–**d4**, respectively are the amplified images of squared areas in **a3**–**d3**. Arrowheads show positive TfR, and ferritin. Bar in **a1**, **a3**, **b1**, **b3**, **c1**, **c3**, **d1** and **d3** is 100 μm, and bar in **a2**, **a4**, **b2**, **b4**, **c2**, **c4**, **d2** and **d4** is 25 μm. **e1**, **e2** Integrated optical density measurement shows that both TfR and ferritin immunoreactivities are increased in the TG mice compared to WT mice. n = 3. *p < 0.05 compared to WT mice

Iron uptake is predominantly mediated via the TfR/Tf system. To examine whether increased IRP1 affects the expression of proteins responsible for iron import and storage, we analyzed TfR, Tf and ferritin expression in N171-82Q mice and age-matched WT mice. By immunohistochemistry, TfR and ferritin immunoreactivities were detected in the 12-week-old N171-82Q mice brain. TfR immunoreactivity was noticeably increased in the HD mice striatum and cortex compared with the corresponding control WT mice (Fig. 5a, b, e), suggesting elevated iron uptake might result from up-regulated IRP1 and TfR in the N171-82Q mice. Surprisingly, the iron storage protein ferritin was not reduced but increased in the N171-82Q mice striatum and cortex (Fig. 5c–e).

**Fig. 6:**
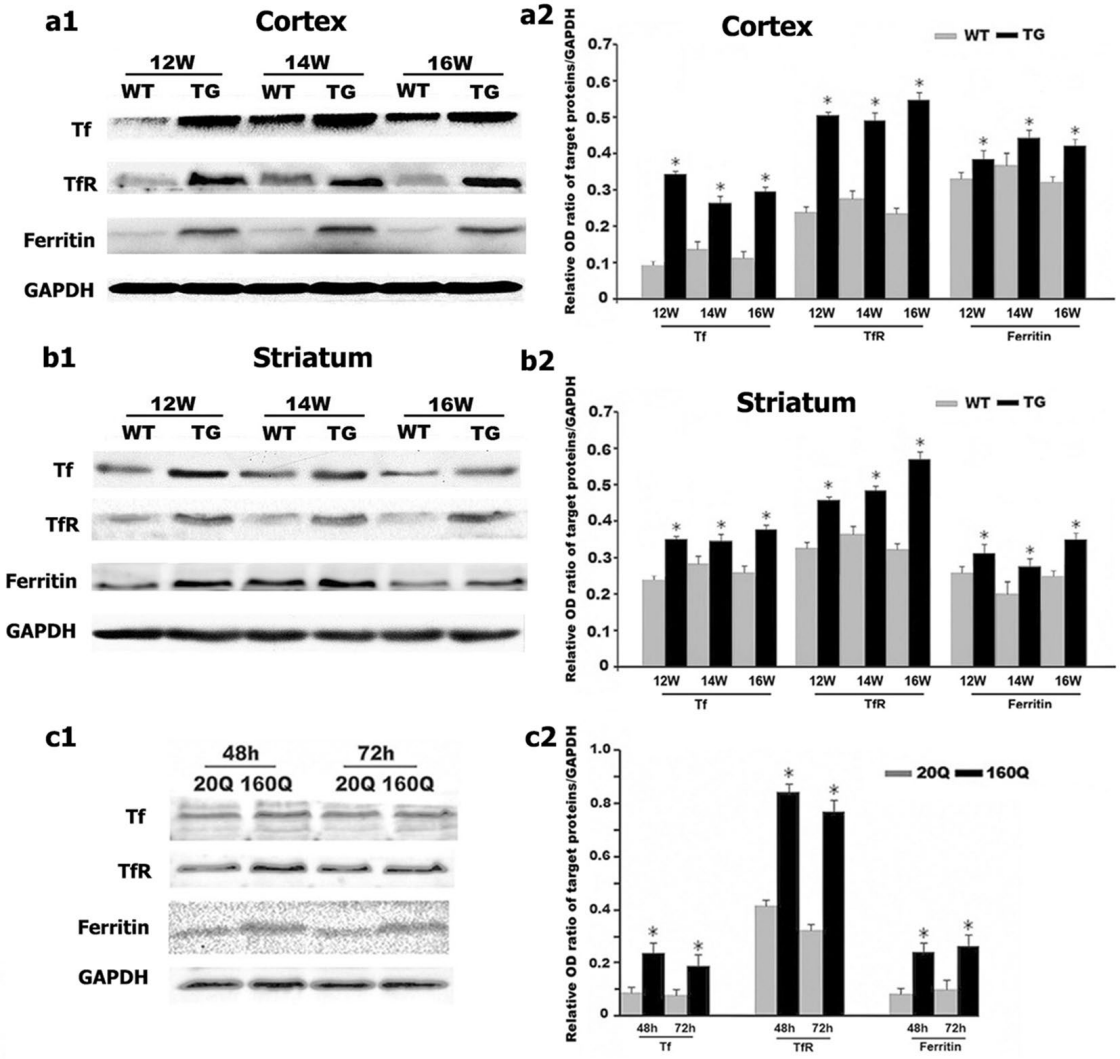
mHTT increases the protein levels of Tf, TfR and ferritin. **a1**–**c1** Tf, TfR, and ferritin protein expressions were detected by Western blotting both in TG mice brain (**a1** cortex; **b1** striatum) and in 160Q HEK293 cells at 48 h or 72 h after transfection (**c1**). **a2**–**c2** Quantitative representation of Tf, TfR, and ferritin band intensity normalized to GAPDH. Tf, TfR, and ferritin protein levels are increased both in the cortex and striatum of TG mice and in 160Q HEK293 cells compared to controls. n = 4. *p < 0.05 compared to WT mice, or 20Q HEK293 cells

**Fig. 7:**
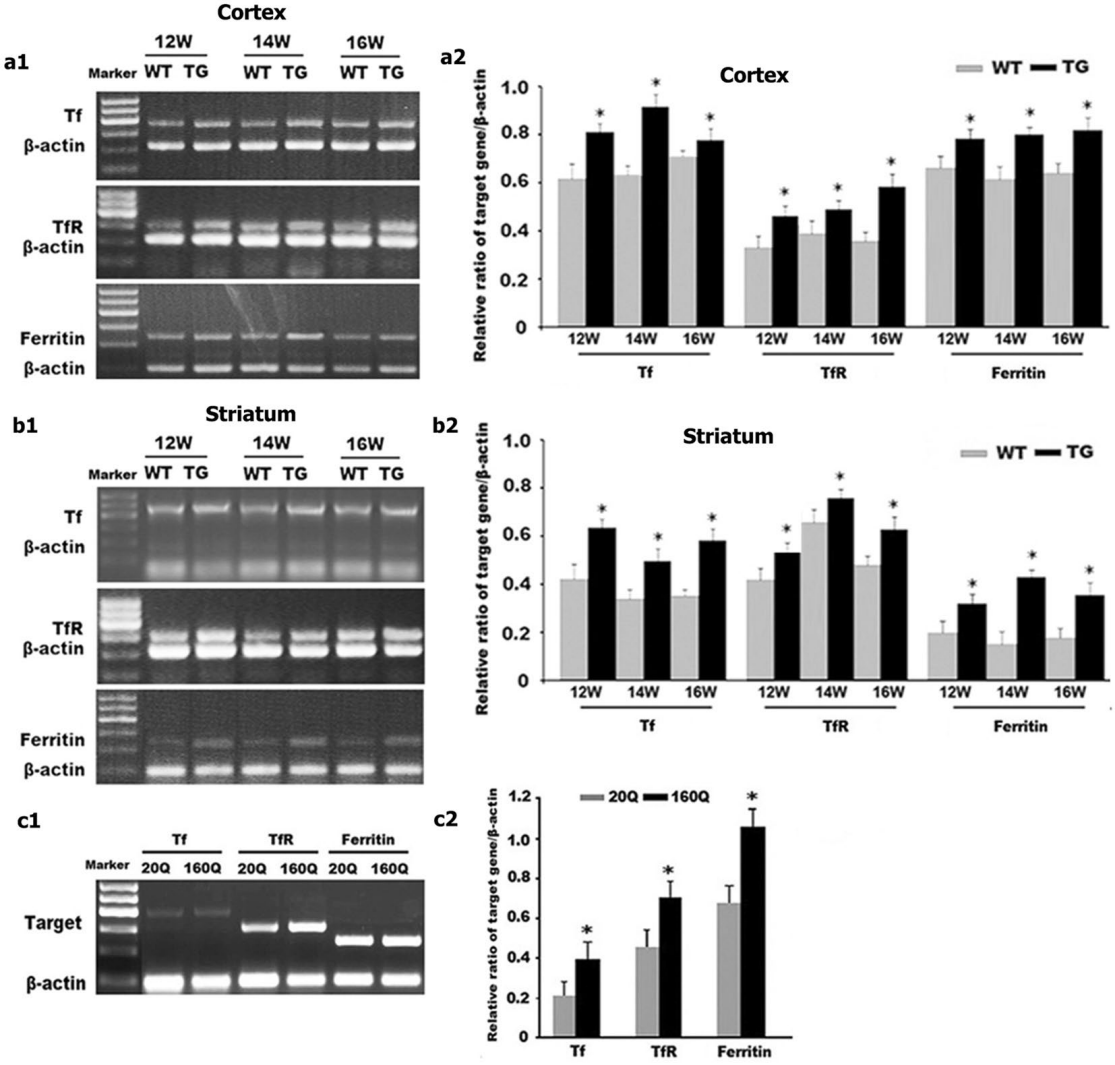
mHTT increases the mRNA levels of Tf, TfR and ferritin. **a1**–**c1** Tf, TfR, and ferritin mRNA levels were detected by RT-PCR both in TG mice brain (**a1** cortex; **b1** striatum) and in 160Q HEK293 cells at 72 h after transfection (**c1**). **a2**–**c2** Quantitative representation of Tf, TfR, and ferritin band intensity normalized to β-actin. Tf, TfR, and ferritin mRNA levels are increased both in the cortex and striatum of TG mice and in 160Q HEK293 cells compared to controls. n = 4. *p < 0.05 compared to WT mice, or 20Q HEK293 cells

We further detected the protein and mRNA expression of TfR, Tf and ferritin in the HD mice by using western blot and RT-PCR. The N171-82Q mice at 12-, 14- and 16-weeks old exhibited increased protein expression and mRNA level of TfR and ferritin in both striatum and cortex compared to age-matched WT controls (Figs. 6a, b, 7a, b). It is noteworthy that Tf, the iron carrying protein, presents extracellular and not directly regulated by neuronal IRP1, was also found to be increased in protein (Fig. 6a, b) and mRNA (Fig. 7a, b) level.

Additionally, we examined the expression of Tf, TfR and ferritin in cultured cell lines. Consistently, there was a significant increase in the protein levels (Fig. 6c) and mRNA levels (Fig. 7c) of Tf, TfR, and ferritin in 160Q HEK293 cells compared to 20Q HEK293 cells. These results suggest that mHTT might disturb iron homeostasis by affecting the expression of iron regulation proteins including IRP1 and Tf. Overloaded iron thus could trigger compensatory changes in ferritin expression.

## Discussion

Iron in the brain presents two types, heme iron and non-heme iron. The latter mainly including iron stored in ferritin and hemosiderin. Previous MRI studies have suggested that increased iron in the striatum could be a causal factor of the symptoms of HD [15, 16]. Imaging technique has significant potential limitations. Changes in the water content can affect the imaging measurements [29] and mask the changes in iron content [30, 31]. Here, we used DAB enhanced Perls’ stain to assess iron content in N171-82Q mice brain. The technique can enhance the signal from the standard Perls’ stain, which is a histochemistry method to detect non-heme iron. The ferric ferrocyanide product can be intensified by allowing it to catalyze the oxidation of DAB in the presence of hydrogen peroxide [32]. We found that the 12-week-old N171-82Q mice displayed intense iron staining in striatum and cortex, although they had not shown any HD symptoms yet. In initial stages of HD, there is gradual progressive degeneration of the striatopallidal white matter projections, resulting in compromised connectivity between the striatum, globus pallidus (GP), and substantia nigra (SN). Neurons also progressively degenerate in the cortex, hypothalamus, and hippocampus; specifically the large pyramidal projection neurons are selectively lost, resulting in atrophy throughout the entire brain by late-stage HD [33]. Iron accumulation in striatum and cortex of the N171-82Q mice could aggravate the toxicity of mHTT to accelerate disease progression. Normally, iron accumulation occurs in the basal ganglia of all aging mammals, which is linked to cognitive and motor dysfunction in the elderly [13, 34, 35]. Here, the N171-82Q mice showed iron overload since their youth, indicating that iron deposition may be one factor to cause motor and cognitive dysfunction in HD. Iron overload is often suggested being a major cause of oxidative stress in neurons [17]. In HD, mHTT leads to an increased production of reactive oxygen species (ROS) [7, 36, 37]. Iron has potential interactions with mHTT-induced oxidative stress in HD. Aggregates formed by mHTT have also been reported to be iron-dependent centers of oxidative stress [18]. Several studies indicated that iron chelator plays a role in reducing cytotoxicity of mHTT, suggesting iron accumulation could contribute to HD onset. Treatment of the metal-binding compound clioquinol improves HD pathology and ameliorate symptoms in R6/2 mice [38]. Iron chelator deferoxamine-treated R6/2 mice showed gradual improvement in behavioral deterioration [14]. Deferoxamine-treatment decreases the oxidation of oxidative stress probes in HD cell lines [18]. Therefore, further analysis would be important to reveal the mechanisms of iron homeostasis disruption in HD.

Abnormal accumulation of brain iron might result from various compounding factors in neurodegenerative diseases including Alzheimer’s, Parkinson’s and neurodegeneration with brain iron accumulation (NBIA) diseases [39–41]. Here, our results indicate that iron accumulation might be related to disruption of iron homeostasis regulatory machinery in HD mice. Iron homeostasis is strictly controlled by a series of regulators. IRP1, a key protein responsible for cellular iron homeostasis, regulates the translations of the mRNAs of proteins involved in iron storage, influx, and efflux [27, 28]. TfR contain IREs in the 3′-UTR of mRNA, and thus IRP1 can control the expression of iron transport in the cell by binding to IREs and inhibiting degradation of the TfR transcript [19, 21]. In the N171-82Q mice and 160Q HEK293, both IRP1 and TfR are up-regulated in protein and mRNA levels, which might be an important factor to iron overload. Consistent with our findings, Trettel et al. reported the activity of iron pathway is dramatically elevated in cultured striatum cells expressing mHTT. These cells display dominant high TfR protein level and increased uptake of Tf [42]. Nonetheless, Chen et al. reported the decreased levels of IRP1, IRP2, and TfR in 12-week-old R6/2 HD mice, which is a compensatory response to iron accumulation in the brain [14]. Both R6/2 mice and N171-82Q HD mice exhibit progressive motor and cognitive deficits, weight loss, mHTT inclusion formation and striatal atrophy accompanied by ventricular enlargement, but no loss of medium spiny neurons (MSNs) [43]. However, they show some differences in pathology including age of onset and life span and metabolism [44, 45]. R6/2 mice express an N-terminal fragment (exon-1 only) of the human HD gene with 150 CAG repeats and develop a progressive neurological disorder with features similar with juvenile onset HD [46]. R6/2 mice develop loss of body and brain weight at 6 weeks, and develop motor dysfunction at 9–11 weeks-of-age, and die at around 100 days-of-age [46]. In contrast to R6/2 mice, N171-82Q mice, expressing a cDNA encoding a 171 amino acid N-terminal fragment of huntingtin containing 82 CAG repeats [47], have a more delayed disease onset and longer survival, with the phenotype beginning at about 90 days-of-age and average death at around 135 days-of-age [47, 48]. N171-82Q mice also show selective striatal pathology, unlike the R6/2 mice where the striatum and cortex are equally affected [44]. The length of CAG repeat is clearly one of the most important variables that affect pathology and progression of the phenotype in different HD mouse models, although it is by no means the only important variable [44, 45]. In our work, 12-week-old N171-82Q mice displayed higher expression of IRP1, though they had not shown any phenotype. However, 12-week-old R6/2 mice, being at the time of late-stage disease in HD littermates, had a lower level of IRP1 [14]. Thus, we hold the idea that CAG repeat length differences in progression of the phenotype might affect outcomes.

In the present study, notably, we also found that the expression of Tf, without IREs in its mRNA, is elevated in N171-82Q mice. Accordant results were observed in 160Q HEK293 cells. These data support our hypothesis that mHTT increases brain iron level by disrupting iron homeostatic pathways. In the plasma, iron is transported by Tf. To enter the central nervous system, iron must cross the blood–brain barrier and the blood–cerebrospinal fluid barrier with the help of TfR on the luminal surface of the capillary endothelial cells. Iron in the endothelia by endocytosis is then released to the brain interstitial fluid, where iron is mainly incorporated with Tf [49]. Afterwards, neurons can import iron by endocytosis of Tf-TfR complex [50]. As the ligand of TfR, Tf gene expression in the brain is not affected by iron [51], but rather is controlled by transcriptional mechanism. Some transcriptional factors including C/EBP, CRI-BP (central region I binding protein) and COUP-TF (chicken ovalbumin upstream promoter transcription factor) regulate the expression of Tf in neurons [52]. C/EBP and CRI-BP play a positive regulator function and COUP-TF acts as a repressor [52]. In the present work, the mechanism of IRP1 and Tf expression up-regulation induced by mHTT is still unknown. Dysregulation of various transcription factors induced by mHTT is also involved in the HD pathological process [4–6]. Mutant huntingtin alters expression of many genes through the interaction with several transcriptional factors in HD models [4, 6]. The striatal-enriched transcription factor COUP-TF-interacting protein 2 is depleted in the striatum of HD patients [53]. Interestingly, COUP-TF can inhibit Tf gene transcription [52]. In addition, the expression of many important heme-based mitochondrial respiratory chain complexes and Fe-S enzymes are altered in HD striatum [54, 55]. Overall, mHtt appears to have multiple toxic gain-of-function properties that could contribute to HD pathogenesis [56]. Thus, it appears likely that mHTT affects IRP1 and Tf expression by way of disturbing transcription, which needs further investigation. The exact mechanism of the effect of mHTT on their activity remains to be fully elucidated.

Under physiologic conditions ferritin mRNA is actively translated to store excess iron in iron surfeit. Here, we found that ferritin was elevated both in the N171-82Q brain and in 160Q HEK293 cells. The result accords with a previous report that ferritin appeared to be increased in the striatum of early grade HD patients [57, 58]. By grade 2 of HD, ferritin staining was also increased in cortex [57]. Ferritin, having a key function in antioxidant activity, keeps ROS to a minimum by sequestering toxic reagents. Consequently, increased ferritin might be corresponding response to overloaded iron in HD and protect cells from oxidative stress caused by mHTT. Taken together, increased ferritin could also serve as a protective response against iron deposition in HD. Further investigation about its role in HD should be performed.

In conclusion, our data indicate that mHTT upregulates the expression of iron regulatory proteins including IRP1, Tf and TfR, which may be one important factor contributing to iron accumulation in HD. These findings could provide a pharmacological strategy to inhibit the expression of IRP1 in the brain for HD treatment.
